# New Insights on Mitochondria-Targeted Neurological Drugs

**DOI:** 10.3390/biology15030228

**Published:** 2026-01-26

**Authors:** Silvia Lores-Arnaiz

**Affiliations:** 1 Facultad de Farmacia y Bioquímica, Fisicoquímica, Universidad de Buenos Aires, Buenos Aires 1113, Argentina; slarnaiz@ffyb.uba.ar; 2 Instituto de Bioquímica y Medicina Molecular (IBIMOL), CONICET-Universidad de Buenos Aires, Buenos Aires 1113, Argentina

**Keywords:** mitochondria-targeted drugs, mitochondrial function, ETC modulation, ROS scavenging

## Abstract

Aging and neurodegenerative diseases are characterized by common features involving bioenergetics deficiencies, oxidative stress and alterations of calcium buffering. Mechanisms of mitochondrial-targeted drugs include the modulation of electron transport chain and oxidative phosphorylation, the binding to mitochondrial lipids, free-radical scavenging, calcium signaling, and possible effects on mitochondrial biogenesis and dynamics and on the regulation of mitophagic pathways. One of the main sites of action of mitochondria-targeted drugs is the interaction with respiratory chain components. Mitochondrial-targeted compounds such as Mito-Q, and Mito-apocynin have been developed by conjugating triphenylphosphonium (TPP^+^) lipophilic cation group with natural molecules, therefore obtaining promising drugs for reestablishing the correct functioning of the mitochondrial respiratory chain. Stabilization of cardiolipin at the inner mitochondrial membrane by elamipretide or SkQ1 and mitochondria-targeted ROS scavengers can also offer a therapeutic approach to prevent bioenergetic impairment associated with several diseases. In addition, the modulation of calcium signaling can be achieved using both MCU agonists and antagonists representing another mitochondrial target for drug therapies development. Finally, potential strategies for treating neurodegenerative diseases based on the modulation of mitochondrial biogenesis, dynamics and/or mitophagic pathways are discussed.

## Simple Summary

Mitochondria play a central role in energy production, free radical formation and calcium homeostasis, as well as in the modulation of neuronal cell death and survival. In the nervous system, neurons rely almost exclusively on ATP synthesis from the mitochondrial respiratory chain and oxidative phosphorylation to fulfill their energy requirements for neurotransmitter synthesis, release and reuptake. Within the nerve terminals, mitochondria are essential to maintain an adequate synaptic function. Mitochondrial dysfunction is involved in physiological aging and in many neurological diseases. Mitochondria-targeted drugs are based on their lipophilic moieties, the possibility of binding to mitochondrial membrane lipids and/or their ability to act as antioxidants or uncouplers. Possible sites of pharmacological intervention that represent mitochondrial targets are mitochondrial electron transport chain and oxidative phosphorylation, cardiolipin, active oxygen species generation, calcium signaling, mitochondrial biogenesis and dynamics and mitophagic pathways. Insights on the potential therapeutic benefits of mitochondrial-targeted drugs are presented herein in terms of the described effects in animal models and clinical studies. The evaluation of mitochondria as a promising target for development of new therapeutic strategies is discussed, with the aim of preventing mitochondrial dysfunction associated with aging damage and neurodegenerative disorders.

## 1. Introduction

Mitochondria play a central role in energy production, free radical formation and calcium homeostasis. They are also critical regulators of neuronal cell death through apoptotic pathways [1]. Evidence has emerged that mitochondrial dysfunction is strongly implicated in the cell damage associated with neurodegeneration; bioenergetic deficiencies can occur early and contribute at different stages to the pathogenesis of neurological diseases [2]. Thus, therapies targeting basic mitochondrial metabolic processes, free-radical generation, or specific interactions between neurodegeneration-associated proteins and mitochondria appear promising for modifying the onset and progression of aging and brain pathologies [3].

In this review, the key physicochemical and pharmacological features of mitochondria-targeted agents are presented along with current knowledge on specific strategies aimed at modulating the electron transport chain (ETC), binding to cardiolipin, scavenging reactive oxygen species (ROS), acting on calcium signaling and modifying mitochondrial biogenesis, dynamics and mitophagic pathways. New insights on the identification and mechanistic characterization of compounds capable of restoring brain mitochondrial bioenergetics and function are also discussed.

## 2. Mitochondrial Function and Drug Effects

In the central nervous system (CNS), mitochondria exert a pivotal role in neuronal function, being essential for meeting the high energetic demands of neurons, maintaining Ca^2+^ homeostasis, and supporting neurotransmission and synaptic plasticity.

Neurons rely almost exclusively on ATP synthesis from the mitochondrial respiratory chain coupled with the oxidative phosphorylation system to fulfill energy requirements for synaptic function [4]. Therefore, at nerve terminals, mitochondria are essential for supplying the ATP required for neurotransmitter synthesis, release and uptake, for maintaining ion gradients through the plasma membrane and restoring calcium homeostasis at both the pre- and post-synaptic compartments [5]. In addition to the mentioned processes, energy demands are necessary for axonal transport of organelles. Synaptic mitochondria are generated in the neuronal cell body and need to be transported to the axon terminal but also back to the cell body in the retrograde direction. Consequently, an appropriate distribution of mitochondria must be maintained in neurons, particularly in distal regions characterized by high energy demands and substantial calcium fluxes, such as synapses and axonal branches [6]. Retrogradely transported mitochondria represent degenerating organelles that are not metabolically active and need to be retrieved back to the soma to be degraded by mitophagy [7].

Impairment of ATP synthesis due to defective electron transfer functioning, increased oxidative stress, damage to mitochondrial DNA, alterations of calcium buffering and changes in mitochondrial dynamics may contribute to inadequate CNS functioning, as observed in aging and neurodegenerative diseases. Neurodegenerative diseases are characterized by a progressive impairment of neuronal integrity and function leading to cognitive and motor deficiencies. One common mechanism in the progression of nervous system pathologies is mitochondrial dysfunction, which can lead to increased ROS generation, calcium deregulation and ultimately neuronal death. Altered mitochondrial structure and function have been implicated in the onset and progression of neurodegenerative diseases such as Parkinson’s, Huntington’s and Alzheimer’s diseases [8].

The involvement of mitochondria in neurotransmitter synthesis and metabolism has been extensively described. For instance, mitochondria contribute to glutamatergic neurotransmission by the generation of α-ketoglutarate at the TCA cycle and its continuous conversion to glutamate by mitochondrial glutamate dehydrogenase [9]. Glutamate is extensively recycled between neurons and astrocytes in a process known as the glutamate-glutamine cycle [10]. During this cycle, one of the forms of phosphate-activated glutaminase (PAG), an enzyme that converts glutamine—supplied from astrocytes—into glutamate, has been shown to be associated with the inner mitochondrial membrane [11]. The recycling of glutamate is closely linked to brain energy metabolism and is essential to sustain glutamatergic neurotransmission [12]. However, excess glutamate release may cause excitability changes in neurons in neurodegenerative diseases mediated by excitotoxicity, mitochondrial Ca^2+^ overload and ROS generation [13]. In addition, dopamine and other bioamines are oxidized by the activity of monoamino oxidase (MAO), an enzyme present at the outer mitochondrial membrane, with the concomitant generation of H_2_O_2_ [14].

Due to the significant role of mitochondria in neurotransmitter synthesis and recycling, modulation of brain energy metabolism and of mitochondrial ROS production is gaining importance as a target in brain aging and neurodegeneration. New neuroprotective therapies have been studied aimed at preserving mitochondrial integrity and function. Pharmacological drugs may act on different mitochondrial sites, such as for instance mitochondrial electron transport chain, membrane proteins and lipids and mitochondrial DNA [15].

## 3. Characteristics of Mitochondria-Targeted Drugs

Some characteristics and properties of mitochondria make these organelles potential drug pharmacological targets, such as the presence of a proton electrochemical gradient across the inner mitochondria membrane, the inner membrane enrichment in some phospholipids (e.g., cardiolipin) and the machinery for the regulation of calcium homeostasis and cell survival [16]. According to these characteristics, different types of mitochondrial targeting can be achieved. Drug targets of pharmacological value may include the following: (a) metabolic pathways (e.g., the Krebs cycle, fatty acid metabolism, the urea cycle, heme biosynthesis, cardiolipin and lipid biosynthesis and metabolism, ubiquinol and Fe-S centers biosynthesis), (b) transport mechanisms (e.g., voltage-dependent anion channels, the ADP/ATP translocator, mitochondrial carrier proteins, calcium transport, and the mitochondrial permeability transition pore), (c) the system of uncoupling proteins (UCPs), (d) the mitochondrial genome, and (e) outer membrane and inner membranes with their differential composition [17].

**Figure 1:**
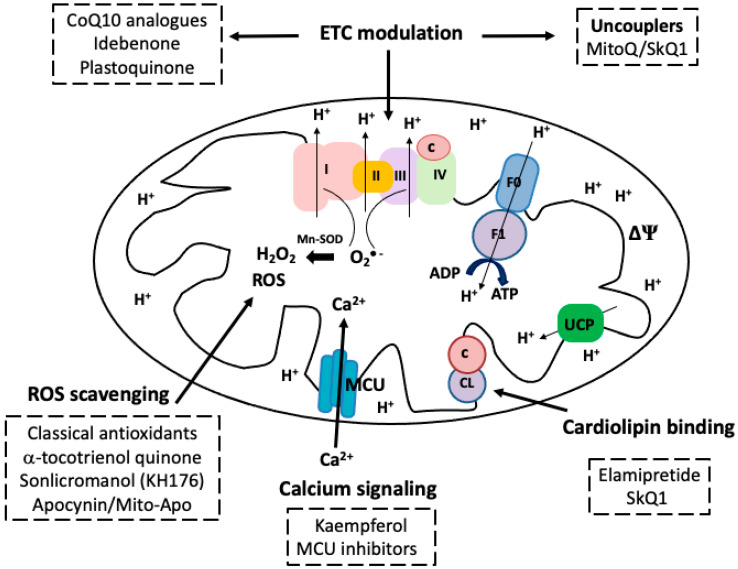
Schematic representation of a mitochondrion showing mitochondrial respiratory chain, oxidative phosphorylation and free radical formation. Electrons are transferred from NADH or FADH_2_ substrates through complexes I–IV of the mitochondrial electron transport chain (ETC). The electron flow is coupled with protons pumping into the intermembrane space, resulting in an electrochemical proton gradient. Re-entry of protons back to the mitochondrial matrix through the F1 subunit of ATP synthase allows the synthesis of ATP. Some sites of mitochondria-targeted drugs are shown. ETC modulation and uncoupling of oxidative phosphorylation, binding to mitochondrial phospholipid cardiolipin (CL), ROS scavenging and calcium signaling are potential targets for restoring mitochondrial function in aging and disease.

Some mechanisms of mitochondrial-targeted drugs imply acting as lipophilic cations that can be easily uptaken and accumulated by mitochondria; others are peptides capable of binding to cardiolipin, and/or imported inside mitochondria via membrane channels [18]. Once inside the organelle, different mitochondrial drugs may act as ETC modulators, antioxidants or uncouplers (Figure 1). In addition, certain compounds can have beneficial effects on restoring mitochondrial biogenesis pathways and/or modulating mitochondrial apoptotic signaling pathways [19].

## 4. Potential Sites of Pharmacological Targeting

Pharmacological approaches have been explored to improve mitochondrial function and reduce the mitochondrial generation of ROS [20]. Potential sites of pharmacological targets to mitochondria are discussed focusing on the following mechanisms of action: (a) ETC and oxidative phosphorylation modulation, (b) binding to mitochondrial lipids (cardiolipin), (c) free-radical scavenging, (d) calcium signaling, (e) effects on mitochondrial biogenesis and dynamics and (f) regulation of mitophagy.

### 4.1. ETC Modulation: Coenzyme Q10 and Analogs

One of the main sites of action of mitochondria-targeted drugs is the interaction with respiratory chain components, such as coenzyme Q. Coenzyme Q10 (CoQ10) is a lipid-soluble molecule, composed of a redox-active benzoquinone ring conjugated to an isoprenoid side chain of 10 isoprenyl units. Two forms of CoQ10 coexist: the oxidized form (ubiquinone) and the reduced one (ubiquinol); both are interconverted allowing a normal functioning of the electron transport at the respiratory chain. Coenzyme Q_10_ exerts a key role as an electron carrier from complex I and II to complex III of the mitochondrial electron transport chain, as well as an antioxidant [21].

Different pathophysiological conditions can be associated with CoQ10 deficiency, which in turn can lead to important consequences for health. Tissues with high energy demands, such as the brain, muscle and kidney, are particularly vulnerable to primary CoQ10 deficiency. In the brain, CoQ10 deficiency can cause ataxia, together with a range of other neurological manifestations [22]. In addition, coenzyme Q deficiency has been described in dopaminergic neurons of patients suffering from Parkinson’s disease, and different therapeutic strategies have been proposed based on supplementation with high levels of Co-Q or with derivative Mito-Q10 [19].

Many drugs have been developed and tested aimed at contributing to the correct functioning of the mitochondrial respiratory chain. Some of them are synthesized from natural products (e.g., Mito-Q, Mito-honokiol, Mito-metformin, Mito-apocynin) and consist of a triphenylphosphonium (TPP^+^) lipophilic cation group attached to an alkyl chain of different length [23]. Among these compounds, mitoQ is a ubiquinone derivative consisting of a ubiquinone moiety attached to the TPP^+^ group. MitoQ can undergo a redox-cycling process between its oxidized (mitoquinone) and reduced (mitoquinol) forms by exchanging electrons with the respiratory chain. Therefore, it can be rapidly regenerated by the respiratory chain after detoxifying ROS, thus acting as an effective antioxidant. MitoQ was shown to protect mitochondria from oxidative damage and prevent apoptosis caused by H_2_O_2_ [24]. However, there is insufficient evidence to support the use of mitochondrial enhancement in patients with neurodegenerative movement disorders, and no significant improvements were found in the motor symptoms of Parkinson’s disease, atypical parkinsonisms or Hungtinton disease patients [25].

Both MitoQ and another conjugate of decyl-TPP^+^ moiety with plastoquinone (10-(6’ plastoquinonyl) decyltriphenylphosphonium or SkQ1) have also been shown to exert uncoupling activity. In fact, MitoQ and SkQ1 rely on ΔΨm to penetrate mitochondria and can increase proton conductance across the inner mitochondrial membrane, therefore being useful to regulate ETC in conditions associated with mitochondrial hyperpolarization [18]. SkQ1 potentiated fatty acid-induced uncoupling of phosphorylation and inhibition of H_2_O_2_ formation [26].

Ubiquinone derivative compounds have been assayed in animal models of neuronal damage. For instance, MitoQ exhibited apparent neuroprotection in cerebral ischemia–reperfusion-induced hippocampal injury in rats, by restoring hippocampal SIRT6 and stabilizating mitochondrial redox status, biogenesis and function [27]. In addition, MitoQ has been shown to improve motor coordination via increasing cerebellar Purkinje cell mitochondria function and reducing Purkinje cell death in a mouse model of autosomal-recessive spastic ataxia of Charlevoix–Saguenay, therefore being a potential therapeutic treatment for pathologies related to motor coordination deficits [28].

Regarding clinical studies for neurodegenerative diseases, mitochondria-targeted drug therapies may be considered auspicious for the treatment of different stages of Parkinson’s and Alzheimer’s diseases [29]. Clinical Phase II trials with MitoQ treatment have been proven to slow the progression of Parkinson’s disease as measured by clinical scores, although they showed no difference between MitoQ and placebo on any measure of Parkinson’s disease progression [30].

Another mitochondrial-targeted compound, named Mito-Apo, generated by conjugating the TPP^+^ group with the non-toxic plant-derived antioxidant apocynin (4-hydroxy-3-methoxyacetophenone), was shown to exhibit neuroprotective mechanisms in a chemically induced Parkinsonian model (MPTP mouse model) [31]. Additional neuroprotective quinones are the synthethic coenzyme Q10 analogues idebenone and plastoquinone, and the endogenous tocopherol-derived quinones, such as vatiquinone and vitamin K [32].

Idebenone (2-(10-hydroxydecyl)-5,6-dimethoxy-3-methyl-cyclohexa-2,5-diene-1,4-dione), a short-chain benzoquinone with structural similarity to coenzyme Q10, can act as a lipophilic electron carrier from respiratory complex II to complex III at the mitochondrial respiratory chain [33]. Regarding idebenone use in human research, the safety, tolerability and pharmacokinetics of high-dose treatment in patients with Friedreich Ataxia have been explored [34,35]. In addition, clinical trials have been performed using idebenone for the treatment of a variety of mitochondria-related disorders; although the drug could be beneficial for the treatment of Leber’s hereditary optic neuropathy [36], results were inconclusive for many of the tested diseases, including Friedreich’s ataxia and Alzheimer’s disease [37]. The lack of success of idebenone in clinical trials for neurodegenerative disorders could be explained by the fundamental difference in the way idebenone affects mitochondrial bioenergetic function in cultured cortical astrocytes, compared with neurons; while idebenone stimulated respiration by astrocytes, cortical neurons were unable to use the drug as a direct mitochondrial electron donor due to the lack of neuronal NAD(P)H:quinone oxidoreductase 1 activity [38].

In addition, the effects of quinones generated during the *in vivo* metabolism of idebenone were evaluated. One specific metabolite, QS10 (6-(9-carboxynonyl)-2,3-dimethoxy-5-methyl-1,4-benzoquinone) was shown to partially restore respiration in cells deficient of complex I or of CoQ [39]. Interesting to note is that another mitochondrial target in relation to modulation of glycolysis and oxidative phosphorylation is the mitochondrial pyruvate carrier (MPC), which mediates the import of pyruvate into the mitochondrial matrix [40]. Compounds that can inhibit MPC have been explored for the treatment of neurodegenerative diseases [41,42].

### 4.2. Binding to Cardiolipin

The dimeric phospholipid cardiolipin is present at the inner mitochondrial membrane and is required for the proper structure and activity of several mitochondrial respiratory chain complexes involved in the generation of ATP [43]. Cardiolipin is particularly rich in unsaturated fatty acids and, therefore, an early target of ROS attack [44]. The main functions of cardiolipin are to help support the spatial organization of mitochondrial cristae and to facilitate the assembly of respiratory super-complexes to allow optimal electron transfer in the respiratory chain [45]. Therefore, stabilization of cardiolipin at the inner mitochondrial membrane can offer a therapeutic approach to prevent bioenergetic impairment associated with several diseases.

Coenzyme Q10 analogue SkQ1 has been shown to be capable of binding to mitochondrial cardiolipin and preventing its peroxidation [46]. In addition, this compound has been shown to be involved in a fatty acid cycling effect resulting in mild uncoupling and the consequent inhibition of the formation of ROS in mitochondrial state 4a [26]. Another compound capable of binding to cardiolipin is elamipretide (SS-31, d-Arg-Dmt-Lys-Phe-NH_2_ peptide), a novel mitochondrial target that has been shown to maintain cellular bioenergetics and prevent ROS-induced cell damage by targeting and stabilizing the cardiolipin-cytochrome c supercomplex [47]. This mitochondrial antioxidant peptide reduces mitochondrial generation of ROS and increases ATP synthesis, thus maintaining the mitochondrial membrane potential. In addition, elamipretide has been shown to prevent the opening of the mitochondrial permeability transition pore and reduce cytochrome c release in response to a high Ca^2+^ overload [48]. Moreover, the treatment with this mitochondrial-targeted peptide elamipretide not only has protective effects against mitochondrial dysfunction but also attenuates surgery-induced pyroptosis and cognitive deficits in aged mice [49]. Also, the *in vivo* studies indicate that LPS-induced memory deficiencies can be attenuated by treatment with elamipretide. This drug can act as a free radical scavenger and inhibit linoleic acid and low-density lipoprotein oxidation [50]. Elamipretide protective mechanisms can also be related to the modulation of BDNF/TrkB signaling; the BDNF/TrkB pathway is critical for synaptic function and plasticity [51] and has also been shown to play a role in regulating mitochondrial biogenesis and efficiency [52], therefore representing another potential target for mitochondrial drug development.

In relation to elamipretide assays in preclinical studies, this peptide has been shown to offer a therapeutic strategy against neurodegenerative diseases, ischemia–reperfusion injury and heart failure in animal research [53,54,55]. In addition, this compound can reduce intracellular Ca^2+^ levels and suppress ROS production both in hearts from diabetic rats and in leukocytes from type-2-diabetes mellitus patients [56]. Moreover, elamipretide has been recently tested in clinical trials to determine its therapeutic usefulness in many conditions characterized by mitochondrial dysfunction [57]. For instance, elamipretide has been shown to increase left ventricular ejection fraction and prevent left ventricular remodeling in animal models of heart failure, as well as to improve cardiac hemodynamics in early-phase clinical trials [58]. In addition, randomized, double-blind, placebo-controlled phase 2 trials showed that daily administration of elamipretide for 48 weeks improved symptoms of Barth syndrome including significant augmentation of skeletal muscle strength and cardiac stroke volume [59]. A more recent study based on randomized, double-blind, placebo-controlled, phase 2 trial evaluated the effects of elamipretide in patients with dry age-related macular degeneration and showed that this drug could preserve photoreceptor function and slow the progression of vision loss in these patients [60].

Regarding elamipretide effects in brain, it is important to note that this drug can cross the blood–brain barrier and target the inner mitochondrial membrane. Previous studies have shown that elamipretide can protect neurons from degeneration in animal models of Alzheimer’s disease [61], Parkinson’s disease [62] and multiple sclerosis [63]. Also, mitochondrion-targeted peptide SS-31 might potentially be a new drug for the early treatment of Friedreich ataxia, as it improved the morphology and function of mitochondria and proved to be efficient in reducing oxidative stress in lymphoblasts and fibroblasts derived from patients [64].

### 4.3. ROS Scavenging and Antioxidant Effects

Mitochondria represent one of the main intracellular sources of ROS generation. Electron transport through mitochondrial enzymatic complexes of the mitochondrial respiratory chain terminates in a four-electron reduction of molecular oxygen to water, catalyzed by the cytochrome c oxidase. Some of the substrate-derived electrons are diverted from the ETC flow and lead to the partial reduction of oxygen, yielding superoxide anion. The highest ROS-producing capacity has been demonstrated for Complex I and Complex III [65].

Some compounds already mentioned such as MitoQ and SkQ1 can scavenge free radicals inside mitochondria and be regenerated by the electron transfer chain [18]. In addition, as discussed before, the novel mitochondrial target peptide elamipretide can also act as an antioxidant, therefore preventing ROS-induced injury [48].

Oxidative stress has been extensively described as involved in aging and age-related conditions such as cardiovascular diseases, chronic obstructive pulmonary disease, chronic kidney disease, neurodegenerative diseases, and cancer [66]. In this context, mitochondria-targeted ROS scavengers can exert an important neuroprotective role in many pathophysiological processes. Besides classical antioxidants like glutathione, ascorbate, N-acetylcysteine or polyphenols, newly synthetized antioxidants have been developed. For instance, the antioxidant effects of a-tocotrienol quinone (ATQ3), a metabolite of a-tocotrienol, have been tested, suggesting that ATQ3 is a potential cellular protectant against oxidative stress and aging. ATQ3 is orally bioavailable, crosses the blood–brain barrier, and has demonstrated clinical response in inherited mitochondrial diseases [67]. Interestingly, Sonlicromanol (KH176), a novel antioxidant agent, was designed to target the thioredoxin (Trx) system. This drug can effectively protect cells from redox balance perturbation by targeting the Trx/Peroxiredoxin system, thus offering a novel approach to the treatment of mitochondrial-related diseases [68].

Different antioxidant compounds have been tested in experimental models of brain pathology. For instance, apocynin has been used as an antioxidant and an inhibitor of NADPH oxidase in pre-clinical models of Parkinson’s disease [69]. However, the efficacy of the antioxidant molecules is strongly dependent on their diffusion through the blood–brain barrier and the limitation of their availability after drug metabolism. In this context, nanoparticles containing mitochondria-targeted antioxidants have been developed and tested in preclinical studies. As an example, biodegradable polyanhydride nanoparticles with apocynin were shown to be effective as an inhibitor of NADPH oxidase in pre-clinical models of Parkinson’s disease and providing protection against oxidative stress-induced mitochondrial dysfunction and neuronal damage in cell systems [70].

More recently, an innovative SC-Nanophytosomes formulation was developed combining elderberry anthocyanin-enriched extract (*Sambucus nigra*) with a lipid-based nanocarrier, which may have potential usefulness for supporting mitochondria-targeted therapies, particularly for pathologies associated with impairment of the mitochondrial respiratory chain activity such as Alzheimer’s and Parkinson’s diseases [71].

At the earliest stages of Alzheimer’s disease, mitochondrial dysfunction leads to oxidative stress in brain cells. The interplay between oxidative stress, mitochondrial dysfunction, and the deregulation of calcium homeostasis induced by misfolded tau and β-amyloid plays an important role in the progressive neuronal loss associated with the disease [72]. In this context, a therapeutic approach based on the combination of classical brain cholinesterase inhibitors with a mitochondria-targeted drug strategy seems promising. A formulation for intranasal administration was developed based on cationic liposomes consisting of soy phosphatidylcholine, cholesterol, and tetradecyltriphenylphosphonium bromide, loaded with antioxidant α-tocopherol in the lipid bilayer and donepezil hydrochloride in the water core. The intranasal administration of liposomes resulted in improvement of learning abilities and a reduction in the formation rate of Aβ plaques in the entorhinal cortex and hippocampus in a model of Alzheimer’s disease transgenic mice (APP/PS1) [73].

### 4.4. Calcium Signaling

Calcium signaling plays a key role during neurotransmitter release; Ca^2+^-triggered synaptic vesicle exocytosis depends on the assembly of the SNARE complex, in which the vesicle-associated v-SNARE protein synaptobrevin interacts with two plasma-membrane-associated t-SNARE proteins, SNAP-25 and syntaxin-1 [74]. Neuronal calcium signaling depends on the calcium distribution in intracellular stores such as the ER, Golgi apparatus and mitochondria, in association with changes in ionic gradients across their membranes. Rapid regulation of intraneuronal Ca^2+^ movement is crucial for synaptic transmission, long-term potentiation or depression [75].

Mitochondria can contribute to calcium homeostasis through their ability to accumulate high amounts of Ca^2+^. Mitochondrial calcium levels can be more than 20 times higher than those in the cytosol due to the following: (a) the presence of mitochondrial juxtapositions with ER-membranes, named mitochondria-associated membranes (MAMs), (b) the presence of an electro-chemical gradient (−180 mV, negative inside) and (c) the existence of a Ca^2+^-selective channel termed the mitochondrial calcium uniporter (MCU) complex, which enables Ca^2+^ accumulation inside the matrix [76,77]. Once inside mitochondria, transient [Ca^2+^]m increases drive mitochondrial ATP production via stimulation of mitochondrial matrix dehydrogenases [78]. However, when calcium overload is high enough to overwhelm mitochondrial capacity of accumulating this cation, MTP pore opening is induced; this process has been shown to be associated with an impaired respiratory function and increased generation of mitochondrial H_2_O_2_ production in mouse non-synaptic brain cortex mitochondria [79].

Mitochondrial calcium uptake mediated by MCU has been shown to play a pathophysiological role in a variety of diseases including stroke and neurodegenerative disorders, therefore making a suitable mitochondrial target for therapeutic intervention. Modulation of MCU activity has been studied using both MCU agonists and antagonists. Regarding the potential neuroprotective effects of MCU agonists, in conditions of energy deficiency, stimulation of MCU might be beneficial as it enhances the activity of the Ca^2+^-dependent matrix enzymes and mitochondrial ATP [80]. To illustrate, kaempferol, a flavonoid compound isolated from *Camelia sinensis*, has shown health benefits; it can act as an MCU stimulant to enhance neurological and neurovascular activity in the normal brain during development and may also provide a new therapeutic strategy to combat excitotoxic damage in amyotrophic lateral sclerosis [81]. On the contrary, upon mitochondrial Ca^2+^ overload, the opening of the MPT pore is induced leading to the triggering of apoptotic cell death pathways, and under these conditions, the future development of small molecule MCU inhibitors may help to prevent MPT pore opening and might be of therapeutic relevance for stroke and neurodegenerative disorders [80].

### 4.5. Regulation of Mitochondrial Biogenesis

Induction of mitochondrial biogenesis may represent an important therapeutic strategy to treat mitochondrial diseases. Mitochondrial biogenesis can help to recover mitochondrial function deficiency by increasing the mitochondrial mass through import of newly synthesized proteins and division of pre-existing mitochondria [82].

Mitochondrial biogenesis can be regulated by different compounds. For instance, the redox cofactor pyrroloquinoline quinone (PQQ) may promote mitochondrial biogenesis and regulate mitochondrial fission and fusion, thus contributing to exert neuroprotective effects in Parkinson’s disease models [83,84]. In addition, mitochondrial energy production and biogenesis may also be enhanced through activation of Sirtuin 1 (Sirt1) by resveratrol or its analogs. Resveratrol, a polyphenol extracted from grape skins, is frequently used as an activator of Sirt 1, which has been proven to induce mitochondrial biogenesis and aerobic capacity in mice through deacetylation and activation of PGC-1α [20]. Neuroprotective properties of resveratrol have been observed in many studies with animal models of neurodegeneration. For instance, resveratrol was effective in preventing memory loss in the amyloid-β protein precursor/presenilin 1 mouse model of Alzheimer’s disease. Long-term resveratrol treatment significantly reduced the amyloid burden and increased mitochondrial complex IV protein levels in the mouse brain, mainly mediated by increased activation of Sirtuin 1 and AMPK pathways [85].

Regarding signaling pathways for mitochondrial biogenesis, PGC-1 plays an important role in regulating mitochondrial function and network organization. Drugs aimed at enhancing its expression and/or activity can be proposed as a therapeutic approach for age-related diseases [86]. For instance, metformin has been shown to induce PGC-1 activity through AMPK activation. It promotes mitochondrial biogenesis and reverses mitochondrial network fragmentation by increasing the expression of Optic atrophy 1 (OPA1) and MFN2, genes related to the fusion processes in Down syndrome cells [87].

Another experimental strategy for increasing mitochondrial biogenesis was based on the developed recombinant human mitochondrial transcription factor A (rhTFAM). TFAM is an essential component of the mitochondrial DNA replication and expression machinery and contains two domains that bind to mtDNA. RhTFAM treatment was able to reduce oxidative stress damage to brain proteins, improve memory performance and increase brain protein expression levels of BDNF and synapsin. Since RhTFAM treatment increases expression of Sirt3 in aged mouse brain, implications of TFAM in aging have also been suggested [88].

### 4.6. Modulation of Mitochondrial Dynamics

Mitochondrial dynamics consists of a complex physiological mechanism of fusion and fission events that allows the recruitment of mitochondria to specific sites of high energy demands. Defects in this machinery have been implied in several diseases including neurodegenerative disorders and cancer [89]. Efforts have been aimed at developing drugs that can restore mitochondrial dynamics. In this context, potential strategies for treating neurodegenerative diseases have focused on targeting the pro-fission protein dynamin-related protein 1 (Drp1) and the pro-fusion protein mitofusin 2 (Mfn2) [37].

The inhibitor of mitochondrial division mdivi-1 (mitochondrial division inhibitor) is a quinazoline derivative capable of interfering with the Drp1 filament assembly, thereby inhibiting mitochondrial fission processes. This inhibitor has been shown to attenuate mitochondrial division by selectively inhibiting the mitochondrial division dynamin and retard apoptosis by inhibiting mitochondrial outer membrane permeabilization in mammalian cells [90]. In addition, mdivi-1 can reduce mitochondrial fragmentation and bioenergetics impairment, prevent deposition of β-amyloid plaques in the brain and improve learning and memory deficits in an AD mouse model [91]. Mdivi-1 has also shown protective effects against the development of multiple sclerosis and reduced inflammation-mediated demyelination in a mouse model of multiple sclerosis called experimental autoimmune encephalomyelitis [92].

A novel Drp1-derived peptide, P110, has been recently designed, capable of selectively inhibiting both Drp1 enzymatic activity and interaction with the mitochondrial adaptor protein mitochondrial fission 1 (Fis1). Administration of P110 led to improved mitochondrial function and decreased cell damage in a model of Parkinson’s disease in culture [93]. P110 could also block MPTP-induced Drp1 mitochondrial translocation and attenuate dopaminergic neuronal loss, dopaminergic nerve terminal damage and behavioral deficits caused by MPTP [94]. Finally, this compound was able to significantly prevent behavioral deficits, and to reduce Aβ accumulation, energetic failure and oxidative stress in the brain of a mouse model of Alzheimer’s disease [95].

### 4.7. Mitophagy

Mitophagy is a form of autophagy that refers to the removal of damaged mitochondria through lysosomal degradation, regulating mitochondrial turnover and neuronal survival; a role of defective mitophagy in neurodegenerative diseases has been proposed, based on the selective degradation of dysfunctional mitochondria [96,97]. Thus, autophagic induction could be used as a therapeutic strategy for neurological illnesses [98].

Mitophagy selectively eliminates impaired and dysfunctional mitochondria mainly mediated by the PTEN-induced kinase 1 (PINK1)/E3 ubiquitin ligase (PARKIN) pathway. PINK1 encodes a serine-threonine kinase with a mitochondria-targeting sequence. When mitochondrial depolarization is detected, PINK 1 is activated and accumulates at the outer mitochondrial membrane, leading to recruitment of PARKIN and the subsequent induction of the autophagic machinery [99].

The PINK1/Parkin pathway is central for the degradation and clearance of damaged mitochondria via mitophagy and can be induced by mitochondria-targeted drugs. Thus, drugs aimed at acting on the interaction between PINK1 and PARKIN can be considered the first approach to protect from mitophagy dysfunction [100].

On the other hand, mitochondria-targeted drugs capable of decreasing mitochondrial membrane potential can activate mitophagy. For instance, it has been described that Mito-Q activates AMPK signaling via depolarization of mitochondria and induction of autophagy, promoting cell survival [101]. In this context, the induction of a mild mitochondrial stress induced by mitochondria-targeting compounds inhibits energy consumption through elevation of AMPK phosphorylation and decreased ATP consumption. In addition, mitochondria-targeted drugs can indirectly inhibit the mTOR (mechanistic target of rapamycin) pathway through inhibition of mitochondrial respiration [102]. Interestingly, evidence suggests a beneficial role of stimulation of autophagy/mitophagy especially in preventing or decreasing the accumulation of alpha-synuclein in the brains of Parkinson’s disease patients [103].

In a recent study by Rosencrans et al. (2025) [104], the effects of two mitophagy activators were characterized: a novel Parkin activator, FB231, and the reported PINK1 activator MTK458. These two drugs can be used in combination to synergistically enhance mitophagy in cell systems and could lower the threshold for mitochondrial toxins to induce PINK1/Parkin-mediated mitophagy, making them potential therapeutic strategies for Parkinson’s disease [104]. However, these data should be analyzed carefully because it was also shown by proteomics studies that FB231 and MTK458 independently induce mild mitochondrial stress, resulting in mitochondrial dysfunction and activation of the stress response [104]. Further studies are needed to investigate therapeutic strategies targeting mitophagy activation in Parkinson’s and other neurodegenerative diseases.

## 5. Conclusions

Impairment of mitochondrial function and imbalance of redox cellular state can lead to a decrease in ATP synthesis and induction of cell death during the onset and progression of several neurological diseases.

Mechanisms of mitochondria-targeted drugs and their implications in neurodegenerative diseases are summarized in Table 1. Pharmacological approaches based on the use of lipophilic antioxidant cations MitoQ and SkQ1 or the peptide SS-31 are promising.

In addition, novel antioxidants are currently tested for neuroprotective effects at the mitochondrial level in the CNS. Targeting calcium signaling, mitochondrial biogenesis, dynamics and/or mitophagic pathways also offer interesting strategies for neurodegenerative diseases and age-related disorders.

Further preclinical and clinical investigations are required to elucidate the signaling pathways modulated by mitochondria-targeted drugs and to identify novel pharmacological targets for mitochondrial-associated diseases.

## Figures and Tables

**Table 1:** Mechanisms of mitochondria-targeted drugs and their implications in neurodegenerative diseases. AD: Alzheimer’s disease; PD: Parkinson’s disease; ALS: amyotrophic lateral sclerosis.

Mitochondria Target	Drug Examples	Neurodegenerative Diseases: Preclinical and Clinical Studies
ETC modulation	Mito-Q Mito-apocynin	Spastic ataxia mouse model (Márquez et al., 2023 [28]) PD patients (Liu and Wang, 2014 [25]; Snow et al., 2010 [30]). MPTP mouse model (Langley et al., 2017 [31])
Coenzyme Q10 analogues	Idebenone	Patients with Leber’s hereditary optic neuropathy (Klopstock et al., 2011 [36]), Friederich’s ataxia and AD (Singh et al., 2021 [37])
Binding to cardiolipin	Elamipretide	Animal models of AD (Manczak et al., 2010 [61]), PD (Yang et al., 2009 [62]) and multiple sclerosis (Fetisova et al., 2017 [63])
ROS scavenging	Apocynin Lipid nanocarriers α-tocopherol-donepezil	Preclinical studies of PD (Ghosh et al., 2012 [69]) Model of AD transgenic mice (APP/PS1) (Vasileva et al., 2023 [73])
Calcium signaling	Kaempferol	Animal models of ALS (Wang et al., 2025 [81])
Mitochondrial biogenesis	PQQ Resveratrol	PD animal models (Zhang et al., 2016 [83]; Lu et al., 2018 [84]) AβPP/presenilin 1 mouse model of AD (Porquet et al., 2014 [85])
Mitochondrial dynamics	Mdivi-1 P110	Mouse models of AD (Baek et al., 2017 [91]) and multiple sclerosis (Li et al., 2019 [92]) MPTP mouse models of PD (Filichia et al., 2016 [94]) and AD (Joshi et al., 2017 [95])
Mitophagy	MitoQ	PD patients (Giordano et al., 2014 [103])

## Data Availability

No new data were created or analyzed in this study. Data sharing is not applicable to this article.

## Abbreviations

AD	Alzheimer’s disease
AMPK	AMP-activated protein kinase
ATQ3	α-tocotrienol quinone
BDNF/TrkB	Brain-derived neurotrophic factor/tropomyosin receptor kinase B
[Ca^2+^]m	Mitochondrial calcium concentration
CNS	Central nervous system
CoQ10	Coenzyme Q10
ΔΨm	Mitochondrial membrane potential
Drp1	Dynamin-related protein 1
ETC	Electron transport chain
LPS	Lipopolysaccharide
MAO	Monoamine oxidase
MCU	Mitochondrial calcium uniporter
mdivi-1	Mitochondrial division inhibitor
MFN-2	Mitofusin 2
MPC	Mitochondrial pyruvate carrier
MPTP	1-methyl-4-phenyl-1,2,3,6-tetrahydropyridine
MPT pore	Mitochondrial permeability transition pore
mtDNA	Mitochondrial DNA
OPA1	Optic atrophy 1
PGA	Phosphate-activated glutaminase
PGC-1α	Peroxisome proliferator-activated receptor-gamma coactivator (PGC)-1alpha
PQQ	Pyrroloquinoline quinone
rhTFAM	Recombinant human mitochondrial transcription factor A
ROS	Reactive oxygen species
Sirt1	Sirtuin 1
SkQ1	10-(6’ plastoquinonyl) decyltriphenylphosphonium
TCA	Tricarboxylic acid cycle
TPP^+^	Triphenylphosphonium
Trx	Thioredoxin
UCPs	Uncoupling proteins
